# PRS-CARV: A summary statistics framework for integrating annotation-informed rare variants to improve polygenic risk prediction

**DOI:** 10.21203/rs.3.rs-10671954/v1

**Published:** 2026-08-31

**Authors:** Yu Zhang, Geyu Zhou, Ming Li, Kelli K. Ryckman, Mitali Ray, Christina Scifres, Nathan R Blue, Alexa Freedman, Jasmina Varagic, George R. Saade, Jane E. Corteville, C. Noel Bairey Merz, Qi Yan, Nianjun Liu

**Affiliations:** 1 Department of Epidemiology and Biostatistics, Indiana University School of Public Health-Bloomington, Bloomington, Indiana, United States of America; 2 Department of Biological Sciences, Purdue University, West Lafayette, Indiana, United States of America; 3 Health Promotion & Development, School of Nursing, University of Pittsburgh School of Medicine, Pittsburgh, Pennsylvania, United States of America; 4 Department of Obstetrics and Gynecology, Indiana University School of Medicine, Indianapolis, Indiana, United States of America; 5 Division of Maternal-Fetal Medicine, Department of Obstetrics and Gynecology, University of Utah Health, Salt Lake City, Utah, United States of America; 6 Department of Preventive Medicine, Northwestern University Feinberg School of Medicine, Chicago, Illinois, United States of America; 7 National Heart, Lung, and Blood Institute, Bethesda, Maryland, United States; 8 Department of Obstetrics and Gynecology, Eastern Virginia Medical School, Norfolk, Virginia, United States of America; 9 Department of OB/GYN-Maternal Fetal Medicine University Hospitals Cleveland Medical Center, Cleveland, Ohio, United States of America; 10 Barbra Streisand Women’s Heart Center, Smidt Heart Institute, Cedars-Sinai Medical Center, Los Angeles, California, United States of America; 11 Department of Obstetrics and Gynecology, Columbia University, New York, New York, United States of America

**Keywords:** polygenic risk score, rare variants, summary statistics, whole genome sequencing, nuMoM2b, lipids

## Abstract

Polygenic risk scores (PRS) primarily rely on common variants because rare variant effects are difficult to estimate without large sequencing cohorts. We developed PRS-CARV, a summary statistics framework that integrates annotation-informed rare variant burden scores with common-variant PRS. In simulations, PRS-CARV consistently outperformed common-variant PRS alone. Applied to whole-genome sequencing data from the nuMoM2b-Heart Health Study, incorporating rare variants improved prediction of lipid traits by 2–11% compared with common-variant PRS. PRS-CARV enables practical integration of publicly available rare variant summary statistics, improving polygenic prediction and supporting broader implementation of precision medicine.

## Background

A central goal of precision medicine is to move disease risk prediction from population-level estimates toward individualized assessment of genetic susceptibility, enabling more personalized approaches to disease prevention and management^1,2^. Polygenic risk scores (PRS) have emerged as one of the key genomic tools supporting this transition by summarizing an individual’s inherited genetic susceptibility to complex diseases and traits^3–5^. Initially developed as research tools, PRS are increasingly being evaluated for clinical risk stratification as evidence supporting their clinical application continues to emerge^6,7^. However, most existing PRS have been developed using common variants identified through genome-wide association studies (GWAS)^3^. Rare variants, although individually uncommon with minor allele frequencies less than 1%, often have substantially larger effect sizes and contribute importantly to the genetic architecture of complex traits^8,9^. Incorporating rare variants into PRS therefore represents an important opportunity to improve the completeness and accuracy of individualized genetic risk prediction, especially for traits where rare functional alleles play an outsized role.

The importance of incorporating rare variants into PRS has become increasingly evident. Several studies have shown that combining rare and common variants can improve genetic risk prediction and risk stratification across diverse complex traits and diseases, including obesity, cancer, and cardiometabolic traits^10–14^. These findings demonstrate that rare variants provide complementary genetic information beyond common-variant PRS and highlight their potential to improve individualized genetic risk prediction. Recent methodological developments have provided statistical frameworks for incorporating rare variants into PRS, including approaches such as RICE^15^ and RareEffect^16^, which estimate rare variant effects using large sequencing datasets. Together, these methods represent important methodological advances for integrating rare variants into polygenic prediction.

However, a common limitation of existing methods is their reliance on individual-level sequencing data from very large training cohorts. Access to such datasets is often restricted by privacy, logistical, and financial barriers, which limits the broader applicability of these methods. Meanwhile, large-scale sequencing efforts such as the UK Biobank^17^, Million Veteran Program (MVP)^18^, and the All of Us^19^ have generated extensive whole genome and exome sequencing data. Analytical tools, such as SAIGE-GENE^20^, STAARpipeline^21^, and REGENIE^22^ can analyze these data at scale and generate publicly available summary statistics. For example, Karczewski et al. (2022)^23^ analyzed whole-exome sequencing data from the UK Biobank with SAIGE, providing precomputed gene-level burden effect sizes across functional annotations, which are available in GENEBASS. The growing availability of gene-level rare variant association summary statistics across thousands of phenotypes presents an opportunity to incorporate rare variants into PRS without requiring access to individual-level sequencing data.

Building on these recent developments, we introduce Polygenic Risk Score integrating Common and Annotation-informed Rare Variants (PRS-CARV), a method designed to leverage summary statistics of rare variant sets from established resources, such as GENEBASS. The proposed PRS-CARV extends the widely used clumping and thresholding (C+T) method to incorporate gene-based rare variant burdens. In the following, we provide a detailed pipeline for implementing PRS-CARV using GENEBASS summary statistics as the base dataset and illustrate its practical application in the Nulliparous Pregnancy Outcomes Study: Monitoring Mothers-to-Be Heart Health Study (nuMoM2b-HHS)^24^ as the target dataset for lipid traits. To evaluate our framework, we conducted simulation studies using genotype data simulated from reference haplotypes derived from the 1000 genomes sequencing dataset^25^. The results show that incorporating rare variants into PRS of lipid traits improves predictive accuracy compared to common-variant-only models. PRS-CARV thus enhances predictive performance while broadening the applicability of PRS through the integration of annotation-informed rare variants using only summary statistics. By enabling more comprehensive characterization of inherited genetic risk without requiring access to large individual-level sequencing datasets, PRS-CARV provides a practical framework that supports the continued evolution of polygenic risk prediction toward individualized risk assessment in precision medicine.

## Methods

### PRS Using Common Variants

As a baseline, PRS is first constructed only using common variants with the standard clumping and thresholding (C+T) method^26^. This method requires two datasets: a base dataset, from which GWAS summary statistics are obtained, and a target dataset, which contains individual-level genotypes for PRS calculation. From the base dataset, we obtained effect sizes of single nucleotide variants (SNVs) and corresponding P-values. LD-based clumping is applied within genomic windows using a greedy selection algorithm, followed by P-value thresholding based on the discovery GWAS summary statistics (e.g., $P<1\times 10^{-5}$). For individual i in the target dataset, the common variant ${\text{PRS}}_{\text{c}}$ is calculated as

$$PRS_{ci}=\sum_{j=1}^{M}\beta_{j}G_{ij}$$

where M is the number of selected common variants, $\beta_{j}$ is the GWAS effect size for variant j from the base dataset, and $G_{ij}$ is the genotype dosage of variant j for individual i in the target dataset.

### PRS Incorporating Common and Rare Variants

To extend beyond the standard common-variant ${\text{PRS}}_{\text{c}}$, we developed PRS-CARV which integrates both common and annotation-informed rare variants. PRS-CARV incorporates rare variants through gene–annotation burden scores, which summarize the cumulative genetic effects of multiple rare variants within biologically meaningful functional categories. For each gene, rare variants (e.g., MAF<0.01) are aggregate separately by functional annotation category (e.g., loss-of-function [LOF], missense, synonymous) to generate gene-annotation burden scores. For individual i, the burden score for gene g and annotation a is defined as

$$B_{iga}=\sum_{k\in V_{ga}}\omega_{k}G_{ik}$$

Where $V_{ga}$ is the set of rare variants in gene g annotated as a (e.g., a∈{loss-of-function,missense,synonymous}), $\omega_{k}$ is a weight for variant k (e.g., Beta(1,25) weighting to upweight ultra-rare alleles), and $G_{ik}$ is the genotype dosage of rare variant k for individual i.

In the base dataset, summary statistics for gene-annotation burden scores $B_{iga}\left(\beta_{ga}\right)$ can be obtained from large-scale rare variant association studies (RVAS) (e.g., GENEBASS). Following Williams et al. (2024), we used a more lenient significance threshold (e.g., $P<1\times 10^{-3}$) for selecting gene-annotation burden scores, compared to the threshold used for common variants (e.g., $P<1\times 10^{-5}$). The greedy LD-based clumping algorithm is then used to retain approximately independent gene-annotation burden scores. In the target dataset, burden scores are computed using the same variant grouping and weighting scheme, ensuring consistency with the base dataset. These target burden scores are then combined with the base dataset effect sizes $\left(\beta_{ga}\right)$ to calculate the selected rare variant set ${\text{PRS}}_{\text{r}}$ for individual i in the target dataset:

$$PRS_{ri}=\sum_{g,a}\beta_{ga}B_{iga}.$$

Finally, PRS-CARV integrates information from the common variants’ PRS $\left(PRS_{c}\right)$ and the rare variants’ PRS $\left(PRS_{r}\right)$ by fitting a regression model in the target dataset, where both scores are included as predictors to jointly evaluate their contribution to phenotype prediction. The overall workflow of PRS-CARV is illustrated in Fig 1.

Note that for common variants, LD can be estimated using either an ancestry-matched external reference panel (e.g., 1000 Genomes Project^25^) or directly from the target cohort^26^. In contrast, LD between rare variant Burden scores could only be computed within the target data, as these gene-annotation Burden scores are derived from individual-level genotypes and cannot be reconstructed from reference panels currently.

### Simulation Setting

To evaluate the performance of our proposed PRS-CARV framework incorporating both common and rare variants, we designed a realistic simulation study that mimics the distributional and annotation properties observed in real whole-genome sequencing datasets.

### Genotype Simulation

To simulate genotype data that includes both common and rare variants, we first used HAPGEN2^27^, which generates haplotypes based on reference panels. However, because HAPGEN2 is known to distort the allele frequency spectrum of rare variants^28^, we applied RAREsim^29^, a rare-variant-aware simulation tool, to post-process the HAPGEN2 output and adjust rare variant distributions to better reflect empirical data. For this simulation, we utilized reference haplotype and legend files from the RAREsim repository (https://github.com/meganmichelle/RAREsim_Example), which include all monomorphic sequencing bases within the coding regions of chromosome 19 from the 1000 Genomes Project^25^ (hg19 build) for non-Finnish European (NFE) individuals. As HAPGEN2 simulates only biallelic SNVs, indels were excluded from the haplotypes. Variants were annotated using ANNOVAR^30^, following procedures similar to those described in the RAREsim^29^. For polymorphic variants observed in gnomAD, we retained the reference and alternate alleles. Monomorphic positions were annotated using all possible alternate alleles via ANNOVAR’s convert2annovar.pl function. A single alternate allele was then selected probabilistically based on a transition-to-transversion (Ti/Tv) ratio of 2.815, consistent with observed values in exome sequencing data. Functional annotations were derived from ANNOVAR’s refGene database and subsequently categorized into three major groups: loss-of-function (LOF; including stopgain and stoploss), missense (nonsynonymous SNVs), and synonymous (synonymous SNVs). A total of 56,885 individuals were simulated to match the NFE sample size of the gnomAD dataset. Using PLINK2^31^, we filtered the resulting data to retain 56,639 unrelated individuals and 299,289 SNVs across the simulated coding regions. To generate gene-level burden scores of those rare variants (MAF<0.01), we summed the dosages of rare variants within each combination of gene and functional annotation, weighting each variant by a Beta(1,25) function of its allele frequency to emphasize the contribution of ultra-rare alleles, thereby obtaining biologically meaningful burden scores per gene–annotation pair.

### Phenotype Simulation

Quantitative traits were simulated under an additive linear model incorporating both common and rare variant contributions. We randomly selected causal variants from both common variants (MAF > 0.01) and gene-based rare variant sets (as defined by functional annotation). The proportion of causal variants was varied across three settings: 1%, 5%, and 20% of all variants. The corresponding numbers of causal common variants and causal rare variant sets were $J_{c}$ and $K_{r}$, respectively, where $K_{r}$ corresponds to the number of selected gene-annotation (g,a) burden scores. Heritability was partitioned as $h_{\text{c}}^{2}=0.3$ for common variants and $h_{\text{r}}^{2}=0.1$ for rare variants set. Effect sizes were drawn from standardized normal distributions: $\beta_{j}\sim 𝒩\left(0,\frac{h_{c}^{2}}{J_{c}}\right),\beta_{ga}\sim 𝒩\left(0,\frac{h_{r}^{2}}{K_{r}}\right)$. Phenotypes were generated for i-th individual as:

$$Y_{i}=\sum_{j=1}^{J_{c}}G_{ij}\beta_{j}+\sum_{g,a}B_{iga}\beta_{ga}+\epsilon_{i},$$

where $G_{ij}$ denotes the genotype of individual i at the j-th causal common variant, $B_{\text{iga}}$ is the burden score for gene g with annotation a. The residual error term $\epsilon_{i}$ was sampled from a normal distribution such that the total phenotypic variance was 1.

### Polygenic Score Construction and Evaluation

We randomly split the simulated samples into 90% training and 10% testing sets for downstream PRS construction and validation. The training set was used to generate GWAS-like summary statistics by estimating marginal effect sizes of common variants and gene–annotation rare variant burden scores with the lm() function in R. These summary statistics were then applied to the test set for PRS construction using the procedures described in the Methods. We compared three configurations: (i) ${\text{PRS}}_{\text{c}}$, constructed from common variants only, (ii) ${\text{PRS}}_{\text{r}}$, constructed from rare variant only, and (iii) ${\text{PRS}}_{\text{c}}+{\text{PRS}}_{\text{r}}$, combing both common and rare variants. Each simulation scenario was repeated 50 times to assess the stability of model performance. Predictive accuracy was evaluated in the test set as the squared correlation $\left(R^{2}\right)$ between observed and predicted phenotypes.

### Real Data Analysis

We applied our framework to real data using the nuMoM2b-HHS cohort^24^ as the target dataset. Phenotypes of interest included both continuous lipid traits measured in the first trimester—high-density lipoprotein cholesterol (HDL-C), low-density lipoprotein cholesterol (LDL-C), triglycerides (TRIG), and total cholesterol (CHOL)—as well as binary outcomes gestational diabetes mellitus (GDM, diabetes first diagnosed during pregnancy) and gestational hypertension disorder (gHTN), encompassing all forms of preeclampsia and gestational hypertension, including superimposed and postpartum cases). As base data, we obtained GWAS summary statistics from GENEBASS (gs://ukbb-exome-public/500k/results/), which provides single-variant and gene-level association results from 394,841 exome-sequenced UK Biobank participants of primarily European ancestry^23^. While GENEBASS represents a general population, and nuMoM2b-HHS specifically focuses on pregnant women, the large sample size and broad coverage of functional annotations in GENEBASS make it an appropriate and representative base dataset for evaluating PRS-CARV. For continuous traits, we used summary statistics for HDL-C, LDL-C, TRIG, and CHOL. For binary traits, we used summary statistics of type 2 diabetes (T2D) for GDM, and essential hypertension (eHTN) for gHTN.

Because GENEBASS is based on European ancestry participants, we restricted the nuMoM2b-HHS target data to individuals of European ancestry to ensure ancestry-matched analyses. Whole genome sequencing (WGS) was available for 4,244 participants. Genetic ancestry was inferred using SNPweights^32^ v2.1 with the 1000 Genomes Project reference data. Individuals were assigned to European ancestry if their predicted ancestry proportion exceeded 50%, yielding 2,972 individuals. To remove closely related individuals, we applied KING^33^ v2.3.2 to excluded all first- and second- degree relatives^26^, resulting in 2,965 European ancestry participants for downstream analyses. Variants in nuMoM2b were subsequently matched to GENEBASS exome variants based on genomic position and allele alignment, ensuring consistency of variant definitions across the base and target datasets.

For PRS construction, we modeled common and rare variants separately, consistent with the procedures described in the Methods. For common variants, we selected variants with MAF > 0.01 from GENEBASS summary statistics, retaining those with P < 1×10^−5^ and using the reported effect sizes as weights. For rare variants, we leveraged gene-level burden test results, which report three functional annotations per gene: predicted loss-of-function (pLoF), missense|low-confidence LoF (missense|LC), and synonymous. Within the nuMoM2b-HHS WGS data, we calculated burden scores per gene-annotation by summing rare alleles (MAF < 0.01), applying Beta(1,25) weights to upweight rarer variants, consistent with the SAIGE-GENE method used in GENEBASS. For each gene-annotation, the corresponding GENEBASS burden effect size was used to weight the burden score.

We then evaluated predictive performance across four models: (1) M0: covariates only (age at visit 1, BMI at visit 1, 10 principal components (PCs) for genetic ancestry); (2) M1: $\text{M}0+{\text{PRS}}_{\text{c}}$; (3) M2: $\text{M}0+{\text{PRS}}_{\text{r}}$; and (4) M3: $\text{M}0+{\text{PRS}}_{\text{c}}+{\text{PRS}}_{\text{r}}$. For consistency, both ${\text{PRS}}_{\text{c}}$ and ${\text{PRS}}_{\text{r}}$ were standardized to have mean zero and unit variance before inclusion, enabling direct comparison of their contributions. Model performance was assessed using the squared correlation (R^2^) for continuous traits and the area under the receiver operating characteristic curve (AUC) for binary phenotypes.

## Results

### Simulation results

In simulation studies, the proposed PRS-CARV framework, which considers both common and rare variants, achieved the highest predictive performance across all scenarios (Fig 2). Compared with ${\text{PRS}}_{\text{c}}$ constructed from common variants only, ${\text{PRS}}_{\text{c}}+{\text{PRS}}_{\text{r}}$ consistently yielded larger squared correlations (R^2^) between observed and predicted phenotypes, demonstrating the added value of incorporating rare variants into PRS. By contrast, ${\text{PRS}}_{\text{r}}$ alone explained little phenotypic variance, which is expected given that the simulated heritability from rare variants ${(h}_{\text{r}}^{2}=0.1)$ was smaller than that from common variants $\left(h_{\text{c}}^{2}=0.3\right)$. This finding indicates that rare variants make limited contributions when considered in isolation, but meaningfully improve prediction when combined with common variants. We also observed that predictive accuracy decreased as the proportion of causal variants increased (e.g., from 1% to 20%). This pattern arises because the total heritability was fixed in the simulations: spreading the same heritability across a larger number of causal variants results in smaller per-variant effect sizes, making individual variants less likely to reach the GWAS-based selection threshold and thus reducing their contribution to PRS prediction. Taken together, these results demonstrate that PRS-CARV improves polygenic prediction over common-variant PRS alone, and that the benefit of including rare variants persists across different proportions of causal variants.

### Application results

For all four lipid traits, inclusion of the common-variant PRS (M1) substantially improved prediction compared with the covariate-only baseline model (M0; Supplementary Table S1). Rare variants alone (M2) explained little variance, consistent with their lower heritability contribution. Table 1 summarizes the key comparison between M1 and the full PRS-CARV model (M3). Adding rare variants on top of common variants provided further gains in prediction, with relative increases in R^2^ ranging from 2.04% for HDL-C to 11.05% for LDL-C. Importantly, ${\text{PRS}}_{\text{r}}$ remained statistically significant (P < 0.05) across all traits in M3, with consistent positive effect sizes, indicating that rare variants provide predictive information beyond common variants alone. To visualize this added value, we stratified individuals into four groups based on the top and bottom 20% of ${\text{PRS}}_{\text{c}}$ and ${\text{PRS}}_{\text{r}}$. As shown in Fig 3, within strata of similar ${\text{PRS}}_{\text{c}}$, individuals with higher ${\text{PRS}}_{\text{r}}$ consistently exhibited higher lipid levels than those with lower ${\text{PRS}}_{\text{r}}$, underscoring the complementary contribution of rare variants. To characterize the biological architecture underlying PRS construction, we examined the functional annotations of all genetic features contributing to the PRSc and PRSr. For common variants, features were mainly missense or synonymous, whereas the rare-variant PRS—although still dominated by missense and synonymous variants—showed a relatively higher contribution from pLoF variants compared with PRSc. At the gene level, PRSc and PRSr showed limited overlap across lipid traits (Fig. 3C; Supplementary Table S2), with only a modest number of shared genes (HDL-C: 23; LDL-C: 21; TRIG: 15; CHOL: 21). The shared genes largely included well-established lipid-related loci such as APOB, PCSK9, ABCG5, and LIPG, whereas the majority of contributing genes did not overlap between the PRSc and PRSr.

In contrast to continuous lipid traits, rare variants did not provide additional predictive value for pregnancy-related binary outcomes. The covariate-only model showed modest discrimination, with only small AUC gains from adding ${\text{PRS}}_{\text{c}}$ and little contribution from ${\text{PRS}}_{\text{r}}$ (Supplementary Table S3). Table 2 presents the key comparison between M1 and the full PRS-CARV model (M3). Incorporating rare variants on top of common variants produced negligible changes in AUC. and the rare variant coefficients were non-significant (P > 0.05) across all outcomes.

## Discussion

In this study, we introduced PRS-CARV, a framework that integrates both common and rare variants into polygenic risk scores using only summary statistics. Through simulations based on realistic sequencing data, we demonstrated that rare variants contribute little predictive power on their own but provide meaningful improvement when combined with common variants. Application to the nuMoM2b-HHS WGS cohort confirmed these findings: incorporating rare variant burden scores significantly improved prediction of continuous lipid traits, whereas no additional benefit was observed for binary pregnancy-related outcomes.

Our results highlight two important points. First, incorporating rare variants provided complementary predictive information beyond common variants, improving prediction accuracy even when their overall heritability contribution was modest. In the nuMoM2b-HHS cohort, inclusion of ${\text{PRS}}_{\text{r}}$ improved the prediction of lipid traits, yielding relative increase in R^2^ ranging from 2% to 11% compared with ${\text{PRS}}_{\text{c}}$ alone. This finding is consistent with previous studies demonstrating that incorporating rare variants into PRS can improve prediction and risk stratification across complex traits such as cardiometabolic phenotypes^12^. This improvement may reflect differences in the functional and gene-level architecture underlying PRSc and PRSr. When considering genetic features selected for the PRS construction, PRSr showed a relatively higher contribution from predicted loss-of-function variants compared with PRSc. At the gene level, overlap between PRSc and PRSr was limited and most contributing genes were present exclusively in either PRSc or PRSr. Together, these findings suggest that common and rare variants capture complementary biological information, with common variants reflecting broadly distributed small-effect genetic contributions and rare variants highlighting protein-disrupting variation in key metabolic genes^34–36^.

Second, the lack of improvement for binary outcomes such as GDM and gHTN likely reflects a combination of statistical, biological, and methodological factors. On the statistical side, the GDM analysis in GENEBASS included only approximately 500 cases among more than 200,000 participants, limiting power to detect rare variant associations. On the biological side, GDM and gHTN are multifactorial pregnancy-related disorders influenced by complex interactions among genetic, physiological, hormonal, and environmental factors, making their risk less likely to be fully explained by genetic variants alone. This interpretation is consistent with previous studies showing that the predictive contribution of rare variants varies across traits, with the greatest improvements generally observed for phenotypes with stronger rare variant architectures, such as certain cancers^10–14^. In addition, because large-scale rare variant summary statistics for pregnancy-specific outcomes are currently unavailable, we used type 2 diabetes and essential hypertension as proxy phenotypes for GDM and gHTN, respectively. Although these traits share important biological mechanisms, they do not fully capture the pregnancy-specific pathophysiology of GDM and gHTN, which may have further attenuated predictive performance. Together, these findings emphasize that the value of incorporating rare variants into PRS is trait dependent and that larger, pregnancy-specific rare variant association studies will be important for fully evaluating the potential of PRS-CARV for pregnancy-related outcomes.

Compared with prior approaches (e.g., RICE^15^, RareEffect^16^, EPRS^14^), PRS-CARV is unique in its reliance on publicly available summary statistics, avoiding the need for individual-level sequencing data to estimate rare variant effects. This design broadens accessibility, allowing researchers to incorporate rare variant information in cohorts where sample size or data-sharing restrictions preclude traditional rare variant analyses. Importantly, PRS-CARV is intended as a flexible framework. In this work, we implemented PRS construction using clumping-and-thresholding with fixed P-value threshold to preserve sample size and ensure stable estimation, particularly in the context of modest target cohorts. Although data-driven tuning approaches—such as selecting optimal p-value thresholds in a held-out subset—are commonly used in PRS analyses, they necessarily reduce the effective sample size available for evaluation and may yield less stable estimates when the target cohort is limited. Cross-fitting strategies can alleviate this loss of power by allowing all individuals to contribute to both training and evaluation across folds. While such strategies could be naturally incorporated into the PRS-CARV framework, we did not pursue them here, as our primary aim was to demonstrate the feasibility and interpretability of integrating common and rare variant effects using summary statistics. More broadly, PRS-CARV specifies how rare-variant burden effect estimates are incorporated and can be applied in a similar manner to other summary-statistic–based PRS methods, including penalized regression approaches (e.g., lassosum2^37^) and Bayesian methods (e.g., PRS-CS^38^ and LDpred2^39^).

Several limitations warrant consideration. First, our real-data analyses were restricted to individuals of European ancestry, as the available GENEBASS summary statistics are derived from European participants; therefore, the transferability of PRS-CARV to other ancestries remains to be evaluated. More broadly, improving the equity and generalizability of polygenic prediction will require large sequencing efforts in diverse populations and the public release of ancestry-specific rare variant summary statistics. Second, the base summary statistics from GENEBASS were generated from a general population, whereas the nuMoM2b-HHS cohort consists of pregnant women. Although differences in physiological and hormonal states may influence trait-specific genetic effects, GENEBASS remains the largest resource providing high-quality rare-variant summary statistics. Third, our real-data analyses were limited to exonic regions, as GENEBASS currently provides rare-variant association results derived from exome sequencing; consequently, regulatory rare variants in noncoding regions were not considered. As large-scale whole-genome sequencing efforts mature and release rare-variant summary statistics beyond the exome, PRS-CARV can be readily extended to incorporate noncoding variation. Fourth, rare variants were aggregated at the gene-annotation level under an additive burden model, assuming that all variants within a set contribute additively and in the same direction. This simplification may not fully capture allelic heterogeneity, dominance, or interaction effects. Emerging frameworks such as STAARpipeline^40,41^ integrate diverse in silico functional annotations^42^—covering epigenetic marks (e.g., H3K27ac, DNase), evolutionary conservation (e.g., phyloP, GERP++), and protein function predictors (e.g., SIFT, REVEL, CADD)—to provide more adaptive and biologically informed variant weighting. As more large-scale sequencing efforts adopt STAAR-based analyses and release annotation-informed rare variant summary statistics^43^, PRS-CARV can be extended to leverage these richer annotations for improved interpretability and predictive power. Future studies expanding PRS-CARV to multi-ancestry populations, incorporating pregnancy-specific sequencing resources, and utilizing whole-genome, annotation-rich rare-variant summary statistics will further enhance its generalizability, precision, and biological interpretability.

## Conclusions

In conclusion, PRS-CARV provides a practical and flexible framework for integrating rare variants into polygenic prediction using publicly available summary statistics. Our results demonstrate that rare variants provide complementary genetic information that improves prediction for lipid traits beyond common variants alone. As large-scale sequencing resources and rare-variant summary statistics continue to expand, approaches such as PRS-CARV will enable increasingly comprehensive polygenic risk scores by integrating complementary information from both common and rare variants. By making more complete genetic risk prediction broadly accessible through publicly available summary statistics, PRS-CARV represents an important methodological step toward precision medicine, where genomic information increasingly informs individualized disease risk assessment and prevention.

## Supplementary Material

Supplementary Files

This is a list of supplementary files associated with this preprint. Click to download.

- supplementtables.xlsx

## Figures and Tables

**Fig 1.**
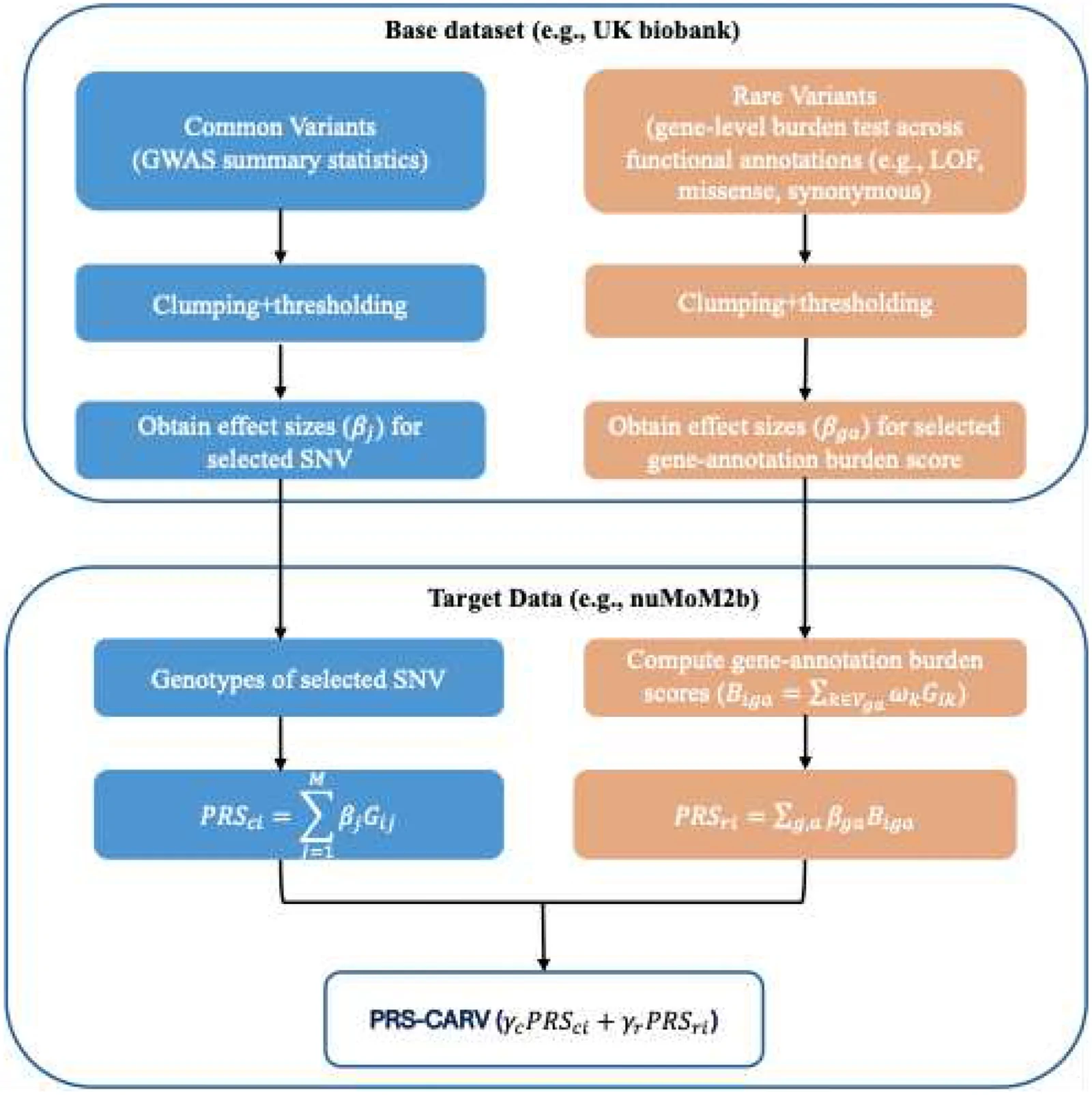
Workflow of the PRS-CARV framework integrating common and rare variants. Common variant summary statistics (left) and rare variant burden test results (right) from large-scale resources such as UK biobank are filtered using clumping + thresholding. Selected variants and burden scores are then matched in the target WGS cohort (e.g., nuMoM2b-HHS) to compute ${\text{PRS}}_{\text{c}}$ and ${\text{PRS}}_{\text{r}}$ for PRS-CARV.

**Fig 2.**
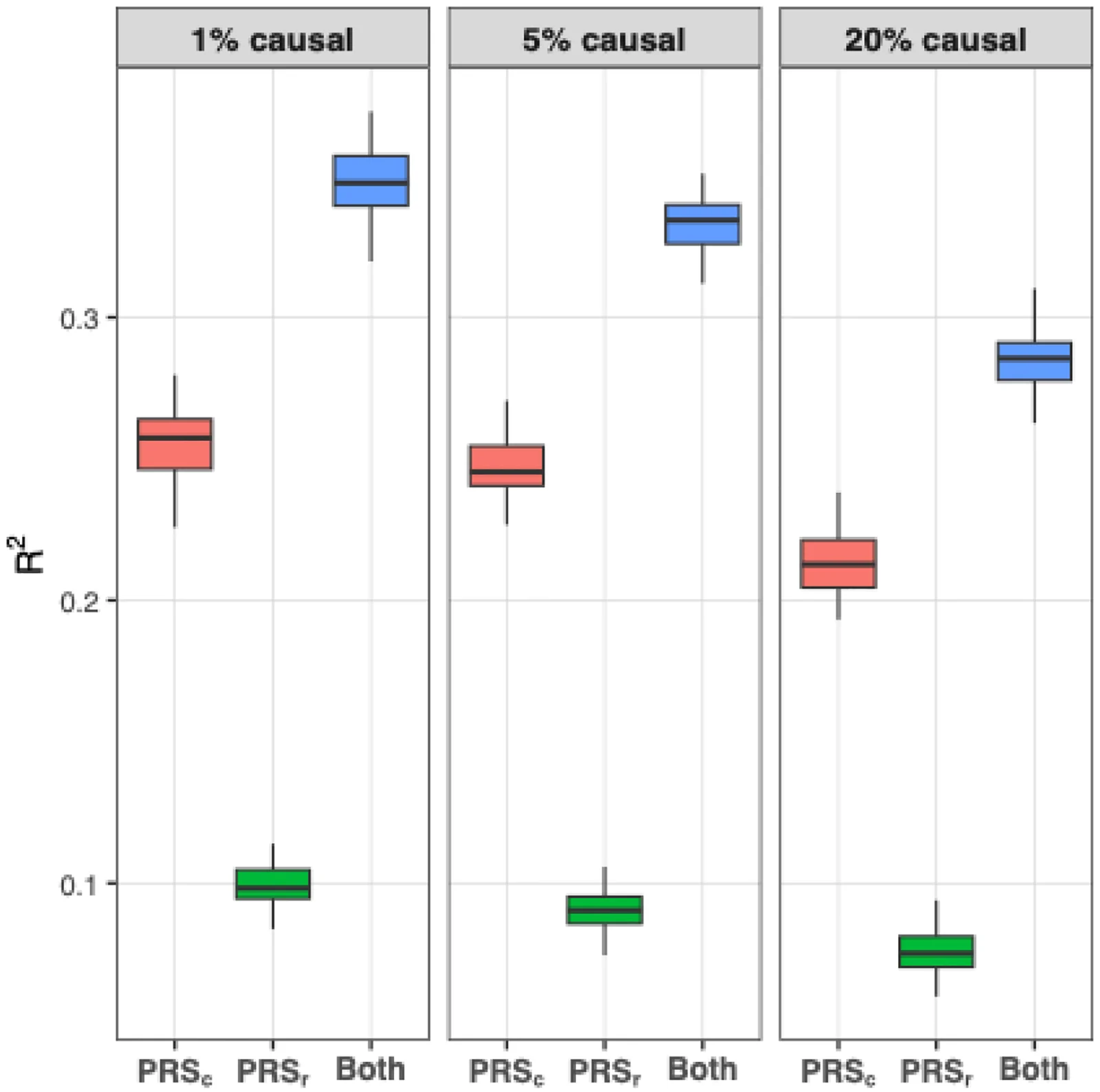
Prediction performance of polygenic risk scores (PRS) across simulation scenarios. Prediction performance of PRS in simulation studies. Boxplots show the squared correlation (R^2^) between observed and predicted phenotypes in test sets across 50 replicates under varying proportions of causal variants (1%, 5%, 20%). Three configurations were compared: ${\text{PRS}}_{\text{c}}$ (common variants only), ${\text{PRS}}_{\text{r}}$ (rare variants only), and Both (common variants + rare variants, corresponding to PRS-CARV).

**Fig 3.**
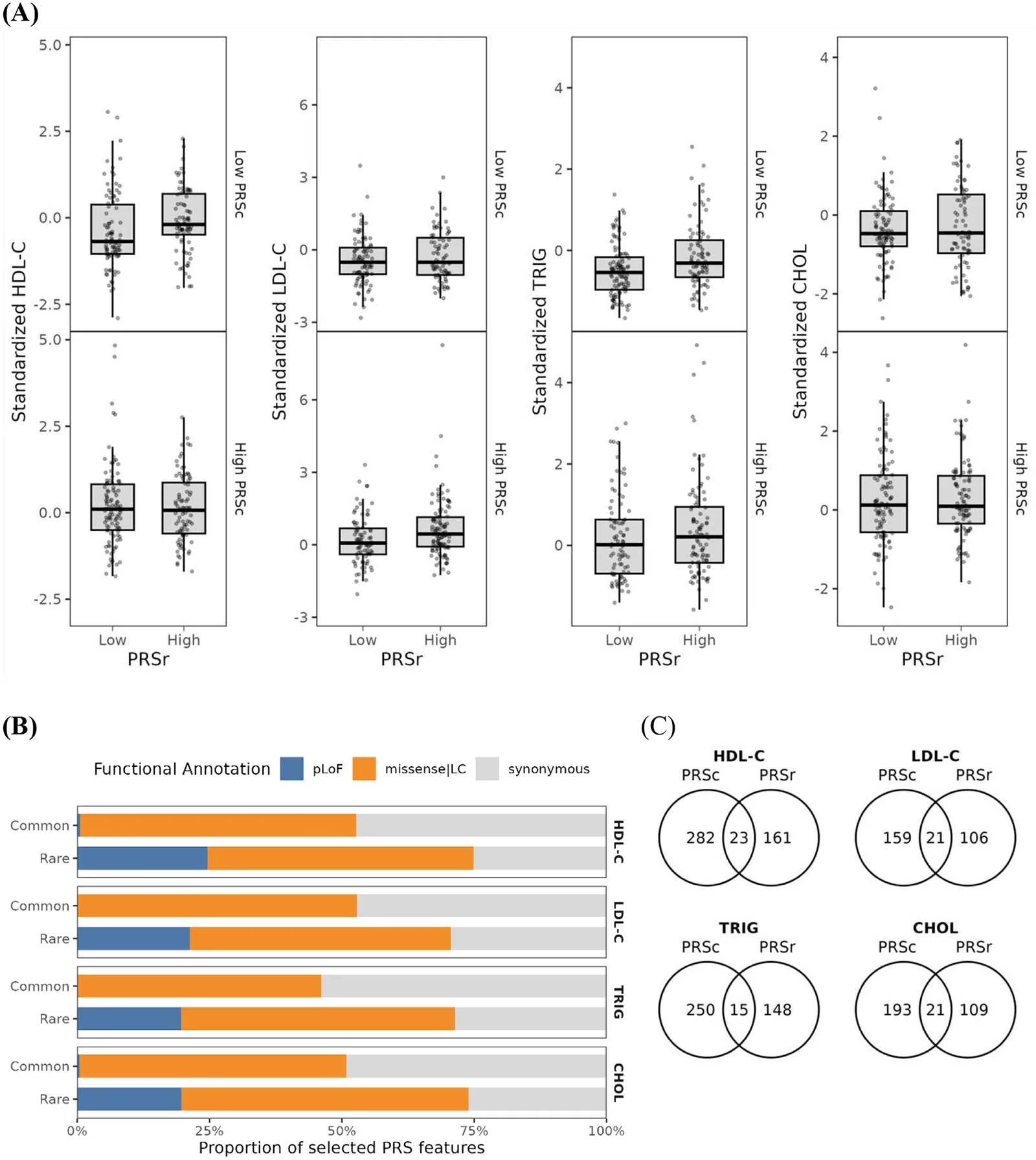
Phenotypic stratification and genetic architecture of polygenic risk scores for lipid traits (HDL-C, LDL-C, TRIG, CHOL). (A) Stratified distributions of lipid traits by common-variant (PRSc) and rare-variant (PRSr) polygenic risk scores. Individuals in the lowest and highest PRS quintiles (bottom and top 20%) were selected to define low and high PRS groups. Boxplots display standardized lipid trait values (z-scores), with overlaid points representing individual observations; (B) Functional composition of the genetic features contributing to PRSc and PRSr across lipid traits; (C) Venn diagrams illustrating shared and unique genes contributing to PRSc and PRSr for each lipid trait.

**Table 1.** Performance of ${\text{PRS}}_{\text{c}}$ (M1) and PRS-CARV (M3) for continuous lipid traits in nuMoM2b-HHS

Trait	$R^{2}(M1)$	$R^{2}(M3)$	$R^{2}((M3-M1)/M1)$	$\beta_{c}(M3)$	$\beta_{r}(M3)$	$P_{r}(M3)$	N
HDL-C	0.1767	0.1803	2.04%	0.2052	0.0605	0.0016	2282
LDL-C	0.0880	0.0978	11.05%	0.2480	0.0992	<0.0001	2277
TRIG	0.1161	0.1239	6.74%	0.1847	0.0897	<0.0001	2282
CHOL	0.0891	0.0931	4.47%	0.2123	0.0635	0.0016	2282

**Table 2.** Performance of ${\text{PRS}}_{\text{c}}$ (M1) and PRS-CARV (M3) for binary pregnancy-related outcomes in nuMoM2b-HHS.

Trait	AUC(M1)	AUC(M3)	AUC((M3-M1)/M1)	$\beta_{c}(M3)$	$\beta_{r}(M3)$	$P_{r}(M3)$	$N_{\text{case}}$	$N_{\text{con}}$
GDM	0.7022	0.7023	0.02%	0.1737	0.1064	0.1079	232	2659
gHTN	0.6530	0.6533	0.05%	0.1422	0.0263	0.5204	957	1976

## Data Availability

GWAS summary statistics from GENEBASS are available at gs://ukbb-exome-public/500k/results/. Access to the nuMoM2b-HHS individual-level whole genome sequencing and phenotype data is available through dbGaP at dbGaP study ID phs002808.v1.p1. PRS-CARV is implemented as an open-source R package available at https://github.com/yu-zhang-oYo/prs.carv.

## List of abbreviations

AUC: Area under the receiver operating characteristic curve
BMI: Body mass index
C+T: Clumping and thresholding
CHOL: Total cholesterol
eHTN: Essential hypertension
GENEBASS: Gene-level burden association summary statistics database
GDM: Gestational diabetes mellitus
gHTN: Gestational hypertensive disorders
GWAS: Genome-wide association study
HDL-C: High-density lipoprotein cholesterol
KING: Kinship-based INference for Genome-wide association studies
LD: Linkage disequilibrium
LDL-C: Low-density lipoprotein cholesterol
LOF: Loss-of-function
MAF: Minor allele frequency
MVP: Million Veteran Program
NFE: Non-Finnish European
nuMoM2b-HHS: Nulliparous Pregnancy Outcomes Study: Monitoring Mothers-to-Be Heart Health Study
PC: Principal component
pLoF: Predicted loss-of-function
PRS: Polygenic risk score
PRS-CARV: Polygenic Risk Score integrating Common and Annotation-informed Rare Variants
PRSc: Common-variant polygenic risk score
PRSr: Rare-variant polygenic risk score
R^2^: Coefficient of determination
RVAS: Rare variant association study
SNV: Single nucleotide variant
STAAR: Variant-Set Test for Association using Annotation information
T2D: Type 2 diabetes
TRIG: Triglycerides
UK Biobank: United Kingdom Biobank
WGS: Whole-genome sequencing
